# Effect of polyphenols from *Ascophyllum nodosum* seaweeds on the rheology and digestion of corn starch gels and gluten-free bread features

**DOI:** 10.1016/j.heliyon.2024.e27469

**Published:** 2024-03-08

**Authors:** Leticia Montes, Maria Santamaria, Raquel Garzon, Cristina M. Rosell, Ramón Moreira

**Affiliations:** a Department of Chemical Engineering, Universidade de Santiago de Compostela, rúa Lope Gómez de Marzoa, s/n. 15782, Santiago de Compostela, Spain; b Institute of Agrochemistry and Food Technology (IATA-CSIC), C/Agustin Escardino, 7, 46980, Paterna, Spain; c Department of Food and Human Nutritional Sciences. University of Manitoba, Winnipeg, Canada

**Keywords:** Corn starch, *In vitro* digestion, Polyphenols, Rheology, Viscosity

## Abstract

The main objective of this work is to study the effect of polyphenols, from the brown seaweed *Ascophyllum nodosum*, on the structure and digestion behaviour of gels at two corn starch concentrations (1.95 and 5.00% w/w) as well as the structure, color and texture features of crumbs from gluten-free breads. Adsorption isotherms of polyphenols on native and gelled starches were carried out and modelled by means of Langmuir and Henry models, respectively. The formation and characteristics of tested gels were rheologically monitored by means of heating ramp, time sweep at high temperature, cooling ramp and frequency sweep at 25 °C. Elastic modulus values decreased with the presence of polyphenols. Additionally, the polyphenols significantly decreased the digestion rate, measured by both chemical and rheological procedures, and the final concentration of digested starch. Finally, the presence of polyphenols in breads increased the hardness and chewiness values and decreased the cohesiveness and resilience values as well as the crumb hardening during storage.

## Highlights

• Initial temperature of gelatinization depended linearly on sorbed polyphenols.
• Polyphenols addition decreased the elasticity of corn starch gels.
• Digestion constant and final digested starch decreased with polyphenols addition.
• Digestion rates were successfully monitored by rheological measurements.
• Polyphenols increased fresh hardness but delayed hardening during the bread storage.

## 1 Introduction

Gluten-free products are in high demand either due to related pathologies or consumers’ choices [1]. However, a high relationship between type 1 diabetes and celiac disease exists because gluten-free diet is richer in carbohydrates and poorer in protein, fiber, minerals, and vitamins [2]. Because of that, there is a growing interest in modulating the starch digestion, particularly in gluten-free starch-based foods.

Polyphenols are secondary metabolites with many reported benefits associated to their antioxidant power, offering protection against development of cancers, cardiovascular diseases, and diabetes among others [3]. Polyphenols have been targeted as great inhibitors of the starch hydrolysis [4]. Other authors observed that corn starch gels enriched with different polyphenols exhibited a notable influence on their digestibility, which was linked to alterations in pH and the quantity of hydroxyl groups [5]. Whether their effect is due to phenolic compounds interaction with starch [6] or a shared action on starch and enzyme [7,8] is still controversial. Therefore, studies are required to understand the polyphenol action as inhibitors of starch digestibility.

Whatever is the hindering mechanism of the polyphenols, their application in food processing requires to identify any technological impact. It has been reported that polyphenols can generate hydrogen bonds with starch, affecting hydration, gelatinization, and retrogradation properties [9]. In the case of rice starch, the incorporation of tea polyphenols decreases the rheological moduli (storage (G′) and loss (G″)), and gel strength, which has been related to the polyphenols interference with amylose crystallization [10]. Even gels can shift from solid-like to liquid-like viscoelastic behavior at high tea polyphenols concentration. Impact on rice starch has been attributed to ferulic acid and gallic acid, that decrease gelatinization temperatures and enthalpies due to hydrogen bonds or van der Waals forces interaction [11]. Likewise, very limited information exists on their impact into final products. The replacement of starch with whole grain red sorghum flour (with high content in phenolics) to improve gluten-free bread was also proposed [12]. A substitution of 30–40% (w/w) of sorghum flour led to increased specific volume and reduced density and texture, which authors associated to an increase in protein. Other authors investigated the incorporation of polyphenol-rich kiwi extract into gluten-free bread formulations and found that bread enriched with the extract was deemed acceptable by a sensory panel, exhibiting a softer and smoother texture compared to regular gluten-free bread, without going into a detailed analysis of the potential effects on the bread's properties [13].

Literature has mainly reported the benefit of phenolic compounds from terrestrial nature, but less information is available about marine polyphenols [14]. It is known that polyphenols from *Ascophyllum nodosum* seaweeds also have many health benefits due to their high antioxidant capacity, offering protection against development diabetes [3], but no information has been provided about their impact on rheology and bread quality. Therefore, the aim of this work is to study the interaction of seaweeds polyphenols with corn starch and resulting gluten-free breads. For this purpose, the adsorption of polyphenols in native and gelled corn starches were studied, as well as the rheology of starch gels with different polyphenol/starch ratios. Low starch concentrations were used to avoid the use high amounts of adsorbate and to observe the polyphenols-starch interactions more easily. In addition, the digestion of these starch gels will be monitored chemically and rheologically. Finally, the polyphenols will be added to a simple starch system for baking and subsequent analysis of the crumb, texture, and colour of the final product. It is worth mentioning that dilutions (1:1) in the mouth, stomach, and intestine, according to the INFOGEST protocol [15], breads with 50% w/w of moisture give rise to low starch content (around 6%) gels to be digested in the intestine.

## 2 Materials and methods

### 2.1 Materials

Corn starch (CS) (moisture 11.4 ± 0.2 dry basis (% d. b.), amylose content 20.25 ± 2.4% d. b.) was purchased from EPSA (Valencia, Spain). Fresh *Ascophyllum nodosum* seaweeds from Galicia's coasts (NW of Spain) were supplied by a local company. Dry baker's yeast was provided by Lesaffre Group (Valladolid, Spain). Type VI-B α-amylase from porcine pancreas (E.C. 3.2.1.1) (14 U/mg solid) and amyloglucosidase (143 U/mL) purchased from Sigma Aldrich. The rest of ingredients for breadmaking were acquired from the local market. Solutions and standards were prepared using deionized water.

### 2.2 Polyphenols extraction

*Ascophyllum nodosum* seaweeds were air-dried at 50 °C for 180 min. Dried *A. nodosum* (10.0 ± 0.1% d.b.) was ground in an ultra-centrifugal mill and stored at 4 °C. More details about procedure can be found in a previous study [16]. The extraction of polyphenols (PP) from *A*. *nodosum* was performed with water as solvent employing a liquid/solid ratio of 20 during 15 min at room temperature under stirring (700 rpm). Then, the mixture was centrifuged (8000×*g*, 10 min), and the liquid phase was filtered (2–4 μm of pore). Total polyphenol content (TPC) of the extract (2.7 ± 0.1 g/L) was determined [17].

### 2.3 Polyphenols adsorption on corn native starch and gels

From the stock polyphenols solution (2.7 ± 0.1 g/L), several dilutions were prepared to obtain different PP concentrations (0.1; 0.3; 0.5; 1.0, and 2.0 g/L). A 3.0 g/L solution was needed to complete an assay, so the stock solution was slightly concentrated (Concentrator plus, Eppendorf, Germany). To those solutions, native or gelled CS was added at two content (1.95 and 5.00% w/v) and kept stirred in an incubator (New Brunswick, Innova 40, USA) at 25 °C and 200 rpm. CS gels were prepared at 1.95 and 5.00% w/w. The dispersions were heated at 100 °C for 20 min with gentle stirring during the first 60 s and then every 5 min for 10 s. The starch gels were cooled (20 min) up to 37 °C for later analyses. Polyphenols adsorption kinetics were monitored during 3 h following TPC values. Equilibrium between solid and liquid phases was achieved when TPC was invariant between measures elapsed 30 min. Adsorbed polyphenols on CS were evaluated by mass balance. Tests were carried out at least in duplicate. Experimental data were fitted by the Langmuir, Eq. (1), and the Henry, Eq. (2), models.

(1) $$q=q_{\max}\frac{{\left[PP\right]}_{e}\;b}{1+b\;{\left[PP\right]}_{e}}$$

(2) $$q=K{\;\left[PP\right]}_{e}$$

where *q* is the amount adsorbed at equilibrium (g polyphenols/g corn starch, g_PP_/g_CS_), [*PP*]_e_ (g_PP_/L) is the concentration at equilibrium, *q*_max_ is the maximum amount of polyphenols adsorbed and *b* (L/g_PP_) and *K* (L/g_CS_) are parameters of the models.

### 2.4 Rheological properties

CS gels were prepared at 1.95 and 5.00% w/w. For 1.95% w/w samples, the nominal PP/CS ratios were: 0.0, 0.5, 2.5 and 5.0; while for gels at 5.00% w/w these ratios were: 0.0, 0.2, 0.9 and 1.9, adding different amounts of PP solutions of 0.0, 0.1, 0.5 and 1.0 g/L (real values are in Table 1). The rheological characterization was performed with a stress-controlled rheometer (Anton Paar 301, Austria) with plate-plate geometry (50 mm) and a gap of 0.25 mm. Samples were covered with paraffin oil to prevent water evaporation. A pre-shearing test was made during 75 min at 100 s^−1^ to ensure the complete adsorption of polyphenols on starch. Then, a heating ramp from 25 to 90 °C (at 4 °C/min) was carried out followed by a time sweep at 90 °C (30 min), a cooling ramp from 90 to 25 °C (at 3 °C/min) and a time sweep at 25 °C (30 min) at a constant strain of 10% and a frequency of 1 Hz (inside linear viscoelasticity range, LVR). Finally, a frequency sweep from 0.01 to 10 Hz was made at 10% of strain and 25 °C. Temperature was controlled by a Peltier system (±0.01 °C). Tests were carried out at least in triplicate.

**Table 1:** Experimental data of polyphenols adsorption on native and gelled corn starch: concentration of starch ([CS]_0_), initial concentration of polyphenols ([PP]_0_), polyphenols/starch ratio (PP_0_/CS_0_), equilibrium concentration of polyphenols ([PP]_e_) and polyphenols adsorbed on starch (q) and initial polyphenols adsorbed (PP_0_ adsorbed).

[CS]_0_ (% w/w)	[PP]_0_ (g_PP_/L)	PP_0_/CS_0_ (%)	Code	[PP]_e_ (g_PP_/L)		*q*, (g_PP_/g_CS_) (PP_0_ adsorbed, %)	
				Native starch	Gelled starch	Native starch	Gelled starch
1.95	0.09 ± 0.01	0.47 ± 0.01	1.95_0.47	0.00 ± 0.00	0.00 ± 0.00	0.005 ± 0.001 (100 ± 0.0)	0.002 ± 0.001 (100 ± 0.0)
	0.26 ± 0.02	1.33 ± 0.08	1.95_1.33	0.08 ± 0.01	0.04 ± 0.01	0.008 ± 0.001 (70.1 ± 1.8)	0.008 ± 0.001 (83.2 ± 0.4)
	0.49 ± 0.02	2.43 ± 0.09	1.95_2.43	0.25 ± 0.01	0.09 ± 0.01	0.010 ± 0.001 (49.3 ± 1.4)	0.017 ± 0.001 (81.0 ± 0.1)
	0.96 ± 0.02	4.79 ± 0.12	1.95_4.79	0.70 ± 0.01	0.23 ± 0.01	0.011 ± 0.001 (27.1 ± 2.5)	0.033 ± 0.001 (75.9 ± 1.3)
	1.95 ± 0.06	9.79 ± 0.30	1.95_9.79	1.69 ± 0.04	0.47 ± 0.01	0.012 ± 0.001 (13.8 ± 0.5)	0.070 ± 0.001 (75.5 ± 1.1)
5.00	0.09 ± 0.01	0.17 ± 0.01	5.00_0.17	0.00 ± 0.01	0.00 ± 0.00	0.001 ± 0.001 (100 ± 0.0)	0.002 ± 0.001 (100 ± 0.0)
	0.26 ± 0.01	0.49 ± 0.01	5.00_0.49	0.07 ± 0.01	0.07 ± 0.01	0.003 ± 0.001 (74.9 ± 0.8)	0.004 ± 0.001 (72.6 ± 0.1)
	0.44 ± 0.01	0.82 ± 0.01	5.00_0.82	0.11 ± 0.01	0.13 ± 0.01	0.005 ± 0.001 (72.6 ± 0.4)	0.007 ± 0.001 (72.5 ± 0.4)
	0.94 ± 0.01	1.77 ± 0.01	5.00_1.77	0.42 ± 0.01	0.32 ± 0.01	0.009 ± 0.001 (55.1 ± 0.5)	0.013 ± 0.001 (68.5 ± 3.7)
	1.83 ± 0.01	3.44 ± 0.01	5.00_3.44	1.17 ± 0.01	0.49 ± 0.01	0.013 ± 0.002 (35.8 ± 0.8)	0.018 ± 0.001 (65.9 ± 0.3)
	3.17 ± 0.01	5.98 ± 0.01	5.00_5.89	2.55 ± 0.01	–	0.011 ± 0.002 (19.7 ± 0.2)	–

### 2.5 Digestion starch

Starch digestion was measured employing two different methods. With the conventional biochemical method of *in vitro* digestion and with the rheometer determining the viscosity drop with time.

*In vitro* digestibility was analyzed following the method previously described with minor modifications [18]. The amount of gel for each sample was weighed to maintain a constant enzyme/starch ratio of 14 U/mg starch. The enzymes used were porcine pancreatic α-amylase and amyloglucosidase. Glucose was determined using a glucose oxidase–peroxidase (GOPOD) kit (Megazyme, Dublin, Ireland). Starch fractions based on the hydrolysis rate were calculated as glucose (mg) × 0.9 and expressed as glucose content (g/100 g gel) using the method previously described [19]. The rapidly digestible starch (RDS) was determined in the fraction corresponding to the first 20 min of digestion, while the slowly digestible starch (SDS) corresponded to the fraction from 20 to 120 min. Tests were carried out at least in duplicate. The *in vitro* digestion kinetics were fitted by first order equation, Eq. (3) [20],:

(3) $$C=C_{\infty }\left(1-e^{-kt}\right)$$

where *C* is the concentration at *t* digestion time, *C*_∞_ the maximum hydrolysis, and *k* the kinetic constant.

The rheological experiments to determine the starch digestion were carried out using a starch pasting cell (ST24-2D/2V/2V-30) as described previously [18] with some modifications. A solvent trap kit was used to minimize water evaporation. A step at 200 min^−1^ during 30 min at 37 °C was made for the sample homogenization and to ensure the polyphenols adsorption on starch. Then, a temperature sweep was carried out from 37 to 95 °C at 5 °C/min at 100 s^−1^ to gel formation. Afterward, a temperature sweep was made from 95 to 37 °C at 5 °C/min at 10 s^−1^ to achieve the required temperature for the enzymatic hydrolysis. A rest time of 36 s was needed to introduce the α-amylase. A stirring period of 10 s at 100 s^−1^ was performed to facilitate the enzyme-starch contact. Apparent viscosity, μ, at 37 °C was monitored at shear rate of 10 s^−1^ (∼physiological shear rates in the small intestinal [21]). Tests were carried out at least in triplicate.

### 2.6 Breadmaking

Gluten-free bread formulations based on CS (100 g) contained 100% water, 2% dried yeast, 1% salt, and 1% sugar. The tested amounts of polyphenol extracts were (0, 0.2, 0.5, and 1.0%). The kneading process was done in the food processor Mambo 10070 (Cecotec, Spain). Half of the water heated at 85 °C was added to the starch and stirred for 10 min at speed 4 promoting its gelatinization. Sugar and salt were added when the gelled mixture was cooled down to 30 °C. The other half of water was separated in two plastic beakers. The yeast was suspended in one and the polyphenols were dissolved in another, and both were added and mixed for 10 min at 500 rpm. Doughs obtained (50 g) were placed into a mold and fermented in a temperature-controlled cabinet (Salva, Guipúzcoa, Spain) for 1 h at 32 °C and 60% relative humidity. After fermentation, the samples were baked at 180 °C for 20 min in a convection oven (Eurofours, Gommegnies, France). Breads were cooled for 30 min at 25 °C before further analysis.

### 2.7 Bread characterization

Bread characterizations were determined as previously reported with moisture content, crumb image analysis, crumb texture profile analysis (TPA) and color [22].

The moisture content of gluten-free bread samples was determined according to the International Association for Cereal Science and Technology method [23]. Moisture content was expressed as g water/100 g bread crumb. Four replicates were analyzed for each sample.

The crumb image analysis was carried out as reported [24]. Bread slices were scanned using a flatbed scanner (Epson V600 Photo, Epson, Japan) in the RGB (Red-Green-Blue) standard format with 1200 dpi resolution. Image analysis of bread slices was conducted using Fiji Image J software [25]. For image segmentation and determination of crumb grain characteristics, image contrast was improved and then the algorithm “Otsu” was applied. The crumb grain measurements included: cell density (cell/cm^2^), mean cell area (mm^2^), surface porosity (total cell area/total slice area in percentage), and mean cell shape using the circularity factor (0-circle to 1-rectangle). Four slices of bread obtained in two different batches (four breads/batch) were analyzed for each sample.

The crumb texture profile analysis (TPA) was carried out using the TA.XT-Plus Texture Analyses (Stable Micro Systems Ltd., Godalming, UK) equipped with a 5 kg load cell and a 25-mm cylindrical probe. Slices were double compressed to 50% of their original height at a crosshead speed of 1 mm/s and 30 s gap between compressions, providing insight into how samples behave when chewed [26]. Parameters recorded were hardness, cohesiveness, springiness, chewiness, and resilience. All textural parameters were measured at 0 and 48 h, and then the hardening rate was calculated, as the hardness increase regarding the hardness of the fresh bread. Data acquired were the average value of eight replicates.

Crumb color was measured using the CIE-L*a*b* uniform color space using a Minolta colorimeter (Chromameter CR-400/410, Konica Minolta, Tokyo, Japan) after standardization with a white calibration plate employing diffusion illumination 0° viewing angle geometry. The color parameters were L* (lightness), a* (hue on a green to red), b* (hue on a blue to yellow) and Δ*E* (total color difference) = $\sqrt{{\left(\Delta L^{*}\right)}^{2}+{\left(\Delta a^{*}\right)}^{2}+{\left(\Delta b^{*}\right)}^{2}}$ which was used to evaluate perceived color differences between control and samples. Measurements were performed on three slices from each run.

### 2.8 Statistical analysis

The results were analyzed through one-factor analysis of variance (ANOVA), followed by the Duncan test, and considering significant p-values <0.05 (IBM SPSS Statistics, New York, United States). Results were expressed as mean ± standard deviation.

## 3 Results

### 3.1 Polyphenols adsorption in native and gelled corn starch

Table 1 collects the concentrations used in the experiments carried out to determinate the polyphenols adsorption in native and gelled CS. For samples with 1.95% w/w of starch content, the initial concentration of polyphenols ([PP]_0_, g/L) varied from 0.09 ± 0.01 to 1.95 ± 0.06, while for samples with 5.00% w/w, [PP]_0_ reached up to 3.17 ± 0.01 g/L for attaining the saturation point of native starch. PP_0_/CS_0_ represents the mass polyphenols/starch ratio (w/w %) for each system. Therefore, the code of the samples was “X_Y”, where “X” was the starch content and “Y” the corresponding mass polyphenol/starch ratio. Equilibrium polyphenols concentration of solution was [PP]_e_ (g/L) and the polyphenols adsorbed was evaluated by *q* (g_PP_/g_CS_).

**Fig. 1:**
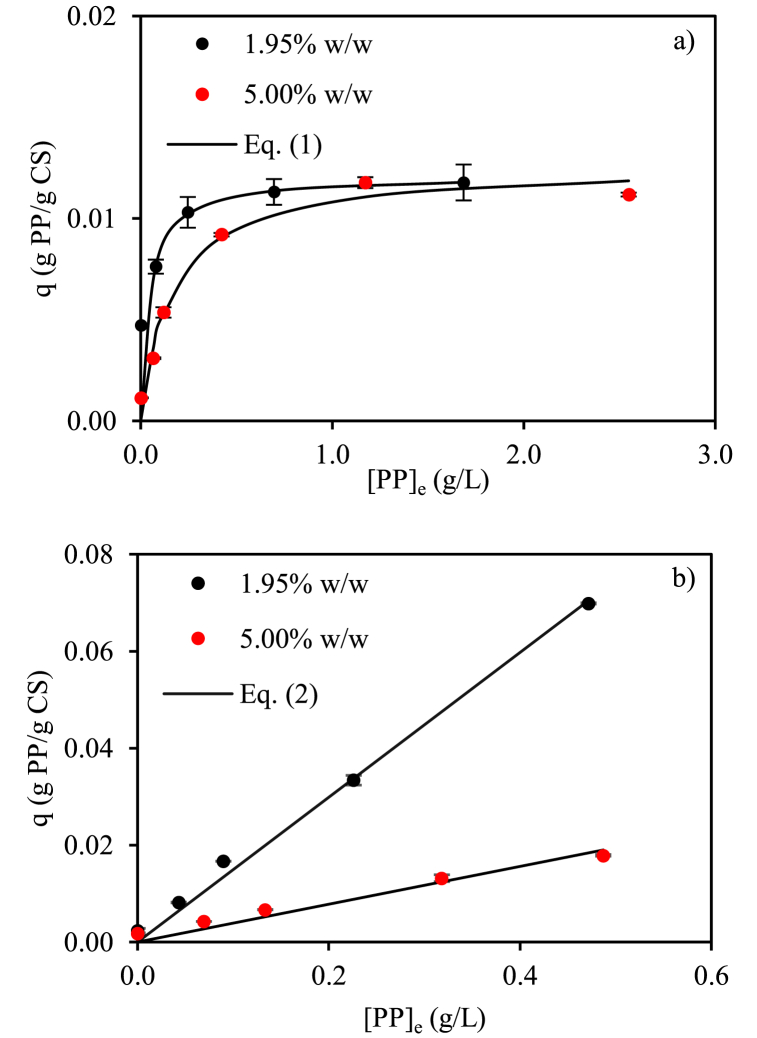
Adsorption of polyphenols at 25 °C on native corn starch (a) and corn starch gel (b) at different starch content. Eq (1) corresponds to Langmuir model and Eq. (2) to Henry model.

Fig. 1 shows the polyphenols adsorption isotherm for native CS at 1.95% w/w and 5.00% w/w (Fig. 1a) concentrations. In the first case, the maximum *q* value (0.012 g_PP_/g_CS_) was obtained above [PP]_e_ of 0.6 g/L. Similar (p > 0.05) maximum *q* value (0.013 g_PP_/g_CS_) was achieved when higher starch (5.00% w/w) concentration was employed, and was constant above [PP]_e_ of 1 g/L. Therefore, the greater the amount of starch, the greater the amount of polyphenols needed to saturate the starch surface. Similar maximum *q* values, independently of starch concentration, allowed to consider that the range of employed starch content was low because the entire surface was available for polyphenols sorption and the contact between starch particles was negligible. The fraction of adsorbed polyphenols, PP_0_ adsorbed, Table 1, decreased with increasing the amount of polyphenols initially present since once saturation was reached, adsorption did not occur. Other authors proposed that between starch and phenolic compounds exist interactions through hydrogen bonds and van der Waals forces depending upon the polyphenols chemical structure [27]. For both systems, the shape of the curve corresponded to a Langmuir isotherm (R^2^ > 0.98) indicating that polyphenols adsorption on the starch surface is physically limited. The parameters (*q*_max_ and *b*) obtained of Eq. (1) are in Table 2.

**Table 2:** Parameters of Langmuir, Eq. (1), and Henry isotherms, Eq. (2).

	Langmuir isotherm (native corn starch)			Henry isotherm (corn starch gel)	
Starch content (% w/w)	*q*_max_ (g_PP_/g_CS_)	*b* (L/g_PP_)	R^2^	*k* (L/g_CS_)	R^2^
1.95	0.012 ± 0.001	21.75 ± 2.07	0.97	0.149 ± 0.003	0.99
5.00	0.013 ± 0.001	6.01 ± 0.22	0.99	0.039 ± 0.001	0.99

Fig. 1b shows the adsorption isotherms corresponding to CS gels at 1.95 and 5.00% w/w concentrations, respectively. When the highest concentrated solution of polyphenols (2 g/L) was employed the maximum *q* values were achieved: 0.070 g_PP_/g_CS_ at low starch content and 0.018 g_PP_/g_CS_ at high content. Conversely to the behavior described for native starch, a linear relationship was achieved between [PP]_e_ and *q* corresponding to Henry's isotherm (R^2^ > 0.99). Therefore, more polyphenols per gram of starch could be retained employing low concentration starch gels. Independently of starch content, polyphenols sorption of starch gels is far from saturation. The parameter values of Henry equation, Eq. (2), are in Table 2. It is worthy to indicate that polyphenols adsorption was notoriously enhanced (up to almost 7-fold) using the starch gel compared to the native starch at the same PP_0_/CS_0_. In fact, the minimum fraction of PP_0_ adsorbed was higher than 65.9% (5.00_3.44) employing starch gels and decreased dramatically to 13.8% (1.95_9.79) with native starch. The gel state increased considerably the available surface area and interactions between starch and polyphenols were also promoted [28,29].

### 3.2 Rheological properties

**Fig. 2:**
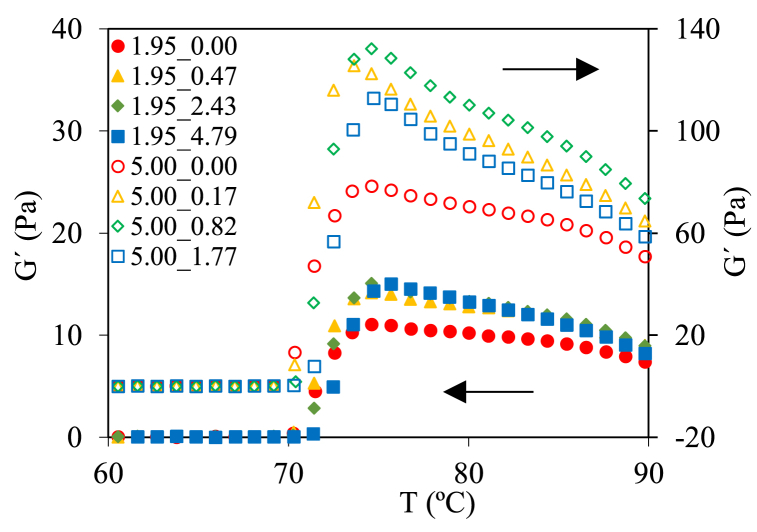
Temperature sweep (from 25 to 90 °C at 5 °C/min, 1 Hz, and 10% strain) for control (only corn starch at 1.95% and 5.00% w/w) and samples with polyphenols. Error bars are not included to improve the clarity of data shown. Legends: code of samples was “X_Y”, where “X” was the starch content and “Y” the corresponding initial mass polyphenol/starch ratio.

Fig. 2 shows the heating ramps from 25 to 90 °C (at 5 °C/min) for tested samples. An increase in the elastic modulus (G') was observed in the presence of polyphenols in comparison to the controls at both starch concentrations. Likely, the interactions between polyphenol-starch chains contribute to form clusters that strengthen the gels [30]. However, Fig. 2, a threshold concentration of polyphenols existed (less than tested concentrations) promoting the elastic character since in the studied range this effect was constant or even caused a gel softening with the increasing of polyphenols content. A delay in the initial temperature of gelatinization (T_0_’) promoted (p < 0.05) by the polyphenols was observed from 69.2 ± 0.3 up to 71.4 ± 0.5 °C for 1.95_0.00 (T_0_’_CS_) and 1.95_4.79, respectively. This effect was also found in 5.00% w/w gels. In fact, a linear relationship (R^2^ > 0.95) between ΔT_0_’, defined as T_0_’ - T_0_’_CS_, and adsorbed polyphenols (*q*) was found.

During heating ramp, the highest value of G' (corresponding to the peak temperature of gelatinization, T_p_) for starch at 1.95% w/w was 11.1 ± 0.3 Pa and increased (p < 0.05) with polyphenols content (up to 15.1 ± 0.4 Pa). In starch gels at 5.00% w/w, the highest value of G' without polyphenols was 78.3 ± 5.9 Pa while for the samples with polyphenols increased noticeably from 112.8 ± 2.9 up to 125.4 ± 4.1 Pa. The presence of polyphenols also slightly delayed the corresponding T_p_. These results could be related to the decrease in water available for starch gelatinization due to the hydrophilic nature of the polyphenols. This trend agreed with the effect observed during the addition of other biopolymers to starches [31]. Above T_p_, G' decreased by the known processes associated to amylopectin crystallites melting and thermal weakening of gel [32], but the polyphenols accelerated this behavior decreasing the differences of G' regarding the samples without polyphenols at 90 °C and consequently the thermostability of gels diminished. This could be related to the filler effect of polyphenols which impaired the aggregation of starch after gelatinization and consequently reduced G' of gels at high temperatures. An attempt was made to relate the increase of T_p_, ΔT_p_, to *q*, similarly to ΔT_0_’, but there was no linear relationship among them. Nevertheless, a linear relationship (R^2^ > 0.98) was found between ΔT_p_ and the PP_0_ in the samples. This may mean that at the beginning of the gelatinization the adsorbed polyphenols interfered the formation of the gel, but they were desorbed along swelling and subsequent breakage of starch.

After the heating ramp, a time sweep was performed at 90 °C for 30 min. A G' drop was observed (p < 0.05) for gels without polyphenols independently of their starch content. The greatest drop occurred during the first 10 min (around 20–25%), reaching a total decrease of approximately 30%. For the samples containing polyphenols, the decrease in G' was lower. In the first 10 min, an average of 15% was achieved, reaching 20–23% on average at the end. This may be related to the crosslinking formed between the polyphenols and the starch, giving a greater elastic character to the gel formed. Evaluating the value of G' in the abrupt change of slope (after 10 min), as ((G'_10'_-G'_30'_)/G'_0_)_CS_ - ((G'_10'_-G'_30'_)/G'_0_)_PP_ *vs* polyphenols/starch ratio, PP_0_/CS_0_ of the starch gels, a power relationship, Eq. (4), was obtained (R^2^ > 0.95):

(4) $${\left(\frac{{G^{'}}_{10^{'}}-{G^{'}}_{30^{'}}}{{G^{'}}_{0}}\right)}_{CS}\;-\;{\left(\frac{{G^{'}}_{10^{'}}-{G^{'}}_{30^{'}}}{{G^{'}}_{0}}\right)}_{PP}=\;0.04\;{\left(\frac{{PP}_{0}}{{CS}_{0}}\right)}^{0.32}$$

where G'_10'_, G'_30'_ and G'_0_ are the elastic modulus at 10, 30, and 0 min in the time sweep at 90 °C.

In the cooling ramp from 90 to 25 °C at 3 °C/min (data not shown), starch gels of 1.95% w/w showed similar behavior. At first (90 °C), gels with polyphenols had higher G' values than control (1.95_0.00) but at the end (25 °C) samples with polyphenols showed lower G' values than control. For starch gels at 5.00% w/w with high polyphenols concentration (5.00_1.77), the behavior was like those observed in gels at 1.95% w/w, but samples with lower polyphenols concentration (<5.00_0.82) showed higher G' values at 25 °C. Likely, low polyphenols/starch ratios (<0.82) were insufficient to interrupt the formation of the final gel. The damping factor (tan δ) in this step followed a similar behavior for all gels, with a decrease from 0.10 to 0.04 without polyphenols, and up to 0.01 with polyphenols. Similar results, lower G' values, for corn and wheat starches when apple polyphenols were added to native starch were previously reported [33]. According to these authors, polyphenols molecules might be inserted among starch molecules, avoiding the intermolecular entanglement, and weakening the intermolecular hydrogen bonds between starches.

In the maturation of the gel (25 °C, 30 min), it was observed that the presence of polyphenols increased noticeably (p < 0.05) the stability of the gels. In fact, at 1.95% w/w of CS gels, G' values of 1.95_0.00 sample increased from 28.0 ± 1.1 to 35.8 ± 0.4 Pa while for gels with polyphenols the increase was less than 2 Pa. For CS gels at 5.00% w/w, similar behaviour was observed with higher changes (from 179.3 ± 5.4 to 205.9 ± 9.1 Pa) in G' values during maturation in gels without polyphenols regarding those formed with polyphenols (i.e.: 192.1 ± 1.5 to 209.2 ± 0.9 Pa for 5.00_0.82). In this step, the tan δ values were similar for all samples studied at both concentrations.

**Fig. 3:**
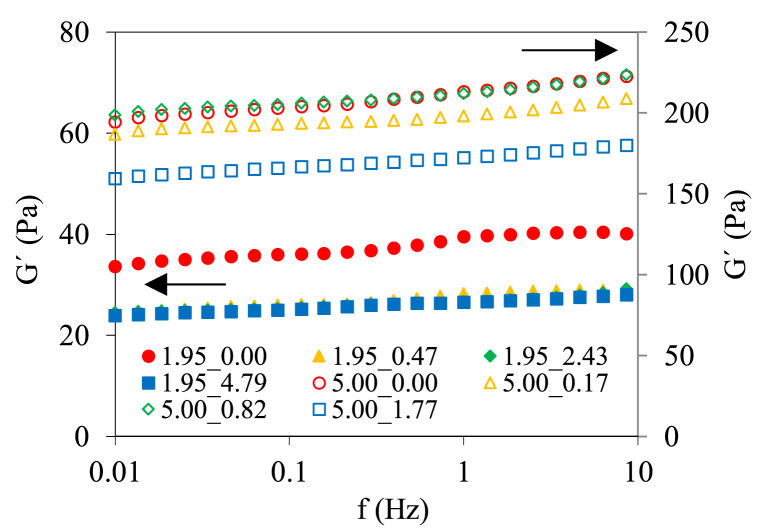
Frequency sweep (from 0.01 to 1 Hz at 10% strain and 25 °C) for control (only corn starch at 1.95% and 5.00% w/w) and samples with polyphenols. Error bars are not included to improve the clarity of data shown. Legends: code of samples was “X_Y”, where “X” was the starch content and “Y” the corresponding initial mass polyphenol/starch ratio.

Fig. 3 shows the frequency sweep from 0.01 to 10 Hz at 10% strain and 25 °C of tested gels. Samples showed a typical behavior of starch gels. The G' values were higher than G'′ values. For gels at 1.95% w/w, a clear effect of polyphenols was observed in the final gel features with lower G' values. However, no significant differences (p > 0.05) were observed with the concentration of polyphenols added, confirming that low concentration was required to interfere in the gel structure and weaken it. In the case of 5.00% w/w starch gels without added polyphenols and low polyphenols content (5.00_0.17 and 5.00_0.82 gels) showed similar behavior, but gels with high polyphenols/starch ratio (>5.00_1.77) showed a significant (p < 0.05) decrease in G' values. It was reported that G' decreased when polyphenols were added to maize, wheat and rice starches and doughs [[33], [34], [35]].

### 3.3 Digestion starch

The digestion kinetic constant (*k*) value for the starch gels without polyphenols (1.95_0.00 and 5.00_0.00) was similar (p > 0.05), Table 3, this could be because the gels are so weak that the structure did not influence this parameter. Independently of starch content of gels, the addition of polyphenols decreased the digestion kinetic constant and the final concentration of digested starch (*C*_∞_). Furthermore, the greater the quantity of polyphenols added, the greater the decrease in *k*. Probably, starch structure changed in the presence of polyphenols, since they could form an "inclusive V-type amylose" complex along α (1 → 4) glycosidic chains with the amylose of the starch [30]. The RDS values corroborated this theory since as polyphenols were added to the gels, less starch was digested. In this way, gels with low starch content without polyphenols (1.95_0.00) a value of 1.62 ± 0.01 g/100 g gel was obtained, while for the gels with the highest polyphenols/starch ratio (1.95_4.79) the value was 0.07 ± 0.01. For the 5.00% w/w gels the same effect was observed. Without polyphenols (5.00_0.00) a value of 2.93 ± 0.13 g/100 g gel was obtained, while for the system with the highest polyphenol/starch ratio (5.00_1.77) decreased up to 0.20 ± 0.02. However, it was observed that SDS values significantly (p < 0.05) increased with polyphenols addition. In any case, the extent of starch hydrolysis, C_∞_, decreased considerably with the added polyphenols in the gels. This result agreed with those found with the addition of gallic acid to rice starch gels [8].

**Table 3:** Parameters of *in vitro* starch gels hydrolysis for 1.95% and 5.00% w/w with different ratios polyphenol/starch by biochemical and rheological methods.

	Biochemical Digestion					Rheological Digestion	
Code	RDS (g/100g)	SDS (g/100g)	*k* (min^−1^)	*k/k*_*oo*_	*C*_∞_ (g/100g)	*k*_*v*_ (min^−1^)	*k*_*v*_*/k*_*vo*_
1.95_0.00	1.62 ± 0.01^a^	0.09 ± 0.01^d^	0.149 ± 0.002^a^	1	1.71 ± 0.01^a^	0.045 ± 0.003^a^	1
1.95_0.47	0.55 ± 0.09^b^	0.80 ± 0.01^a^	0.024 ± 0.004^b^	0.16 ± 0.03^a^	1.43 ± 0.07^b^	0.007 ± 0.002^b^	0.15 ± 0.02^b^
1.95_2.43	0.12 ± 0.01^c^	0.30 ± 0.01^b^	0.013 ± 0.001^c^	0.09 ± 0.01^b^	0.54 ± 0.01^c^	0.002 ± 0.001^c^	0.04 ± 0.01^a^
1.95_4.79	0.07 ± 0.01^c^	0.21 ± 0.01^c^	0.008 ± 0.001^c^	0.05 ± 0.01^c^	0.44 ± 0.01^d^	0.0006 ± 0.0001^d^	0.013 ± 0.001^c^
5.00_0.00	2.93 ± 0.13^A^	0.21 ± 0.03^B^	0.136 ± 0.005^A^	1	3.14 ± 0.16^A^	0.195 ± 0.006^A^	1
5.00_0.17	2.53 ± 0.03^B^	0.50 ± 0.17^A^	0.092 ± 0.015^B^	0.67 ± 0.09^A^	3.02 ± 0.13^A^	0.118 ± 0.004^B^	0.61 ± 0.03^A^
5.00_0.82	0.19 ± 0.02^C^	0.54 ± 0.01^A^	0.012 ± 0.002^C^	0.09 ± 0.01^B^	1.05 ± 0.10^B^	0.006 ± 0.001^C^	0.03 ± 0.01^B^
5.00_1.77	0.20 ± 0.02^C^	0.54 ± 0.01^A^	0.011 ± 0.002^C^	0.08 ± 0.01^C^	1.00 ± 0.02^B^	0.006 ± 0.001^C^	0.03 ± 0.01^B^

Digestion data are reported in g of starch hydrolysed per 100 g of gel. Data are presented as mean ± standard deviation. Data value of each parameter with different superscript letters, in columns, are significantly different (p < 0.05).

On the other hand, the study of starch hydrolysis by rheometry was based on the drop in the apparent viscosity with time. Firstly, a calibration of the apparent viscosity (measured at shear rate of 10 s^−1^) was carried out with gels of starch concentrations from 0.5 to 5.0% w/w dry basis. Eq. (5) was obtained relating the apparent viscosity with starch content (R^2^ > 0.99):

(5) $$C=0.637\;\ln\left(\mu_{d}\right)+4.582$$

where C (% w/w) is the starch concentration and μ_d_ (Pa s) corresponds to the apparent viscosity of starch gel considering the dilution after maleate buffer addition (without enzyme). The effect of dilution had to be considered in the calibration due to viscosity change considerably with the presence of buffers.

**Fig. 4:**
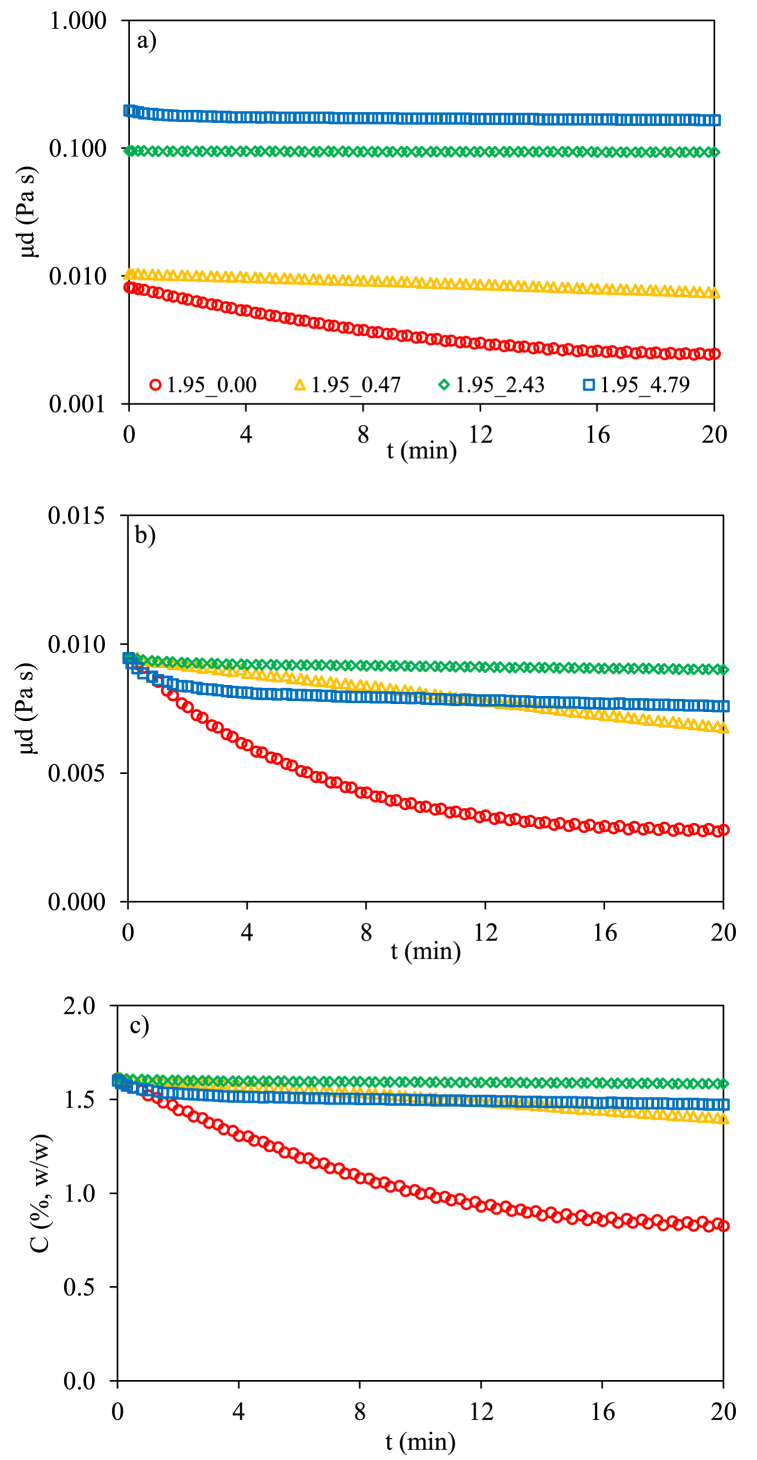
a) The raw apparent viscosity (log scale), b) viscosity curve after applying the corresponding conversion factors, c) the starch concentration curve during digestion as function of time for control (1.95_0.00) and samples with added polyphenols.

In the digestion study, the enzyme was added to the gel diluted in maleate buffer. Immediately, apparent viscosity data were measured in a time sweep step up to achieve a viscosity plateau meaning that starch digestion was finished. The initial apparent viscosity of starch gels increased with added polyphenols, μ_dPO_, (at the highest polyphenols/starch ratio (1.95_4.79) the initial viscosity was 9-fold higher starch gel without polyphenols, μ_dO_ (1.95_0.00). For this reason, a correction factor, given by experimental (μ_dPO_/μ_dO_) was applied to the experimental values for employing adequately the calibration, Eq. (5), in tested gels. Fig. 4a shows the raw apparent viscosity (log scale) curves obtained during starch digestion, Fig. 4b, the viscosity curve after applying the corresponding conversion factors and, Fig. 4c, the starch concentration curve during digestion.

**Fig. 5:**
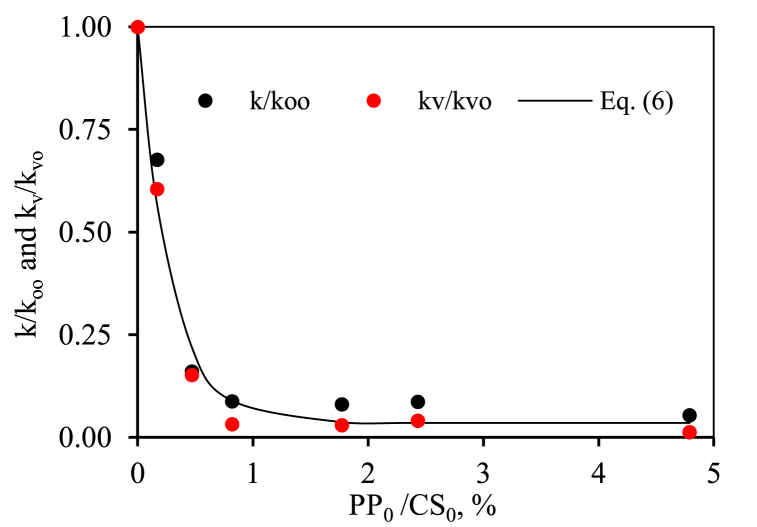
Experimental relative kinetic constants (by biochemical and rheological methods) as function of polyphenols/starch ratios, PP_0_/CS_0_, and proposed model, Eq. (6).

The digestion curves were treated with the same model, Eq. (3), employed in the biochemical method and kinetic constant, *k*_*v*_, values are in Table 3. The results obtained by rheology about the effect of polyphenols on digestion were like those reported by biochemical digestion and confirmed that *k*_*v*_ decreased with the presence of polyphenols. Despite the kinetic constant values obtained by biochemical and rheological methods, *k* and *k*_*v*_, were numerically different (justified because geometry, scale, stirring, homogenization, etc., were different between both experimental setups), when relative kinetic constant, evaluated as *k/k*_*oo*_ or *k*_*v*_*/k*_*vo*_, (using the corresponding starch gels without polyphenols as references) were employed a good agreement was found, Table 3. Fig. 5 shows the relationship between *k/k*_*oo*_ or *k*_*v*_*/k*_*vo*_ and the polyphenols content for starch gels with 1.95 and 5.00 % w/w. Starch digestions were similarly slowed down following an exponential relationship (R^2^ > 0.991), Eq. (6), with the polyphenols/starch ratio present independently of the initial starch content of the gels.

(6) $$\frac{k}{k_{00}}\;or\frac{k_{v}}{k_{v0}}=\frac{0.965}{{\;e}^{\left[3.510\;\left(\;\frac{{PP}_{0}}{{CS}_{0}}\right)\right]}}+0.035$$

### 3.4 Bread characterization

**Fig. 6:**
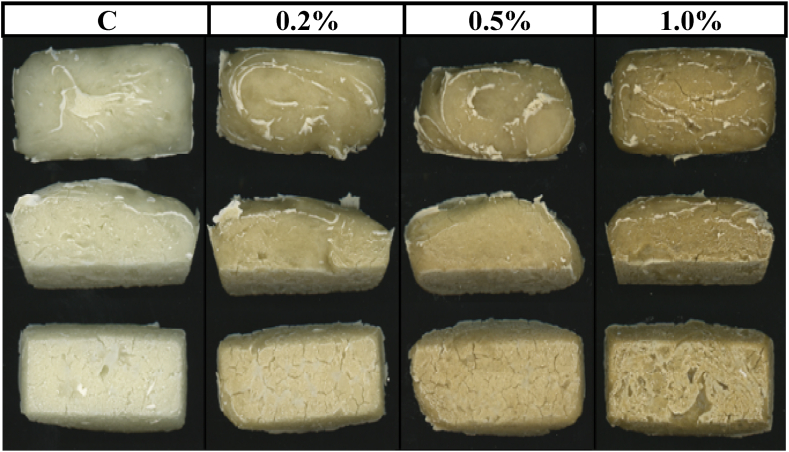
Images of baked bread for control (C) and with addition of 0.2, 0.5, and 1.0% w/w starch basis of polyphenols.

The polyphenols addition, in general, affected the analyzed characteristics (Table 4) as well as the appearance of the gluten-free breads (Fig. 6). The moisture content decreased with the addition of polyphenols (Table 4), but significant (p < 0.05) differences were only found in the samples with the highest percentage of polyphenols (1.0% w/w starch basis). Likely, less hydroxyl groups were available to link water molecules, which could be indicative of starch-polyphenols interactions [36]. The color of the bread was significantly (p < 0.05) affected by the polyphenols added (Table 4). The lightness (L*) decreased linearly (R^2^ > 0.97) with the addition of polyphenols. Parameter a* increased with the addition of polyphenols except for samples with 0.2% w/w starch basis that decreased and b* values increased significantly with the polyphenols added.

**Table 4:** Bread characterization values: Control (C) and polyphenol-enriched variants at 0.2%, 0.5%, and 1.0% w/w starch basis.

Analysis	Storage (h)	C	0.2%	0.5%	1.0%
Moisture (% w.b.)	0	50.46 ± 0.40^a^	50.14 ± 0.40^a^	49.91 ± 0.76^a^	48.57 ± 0.34^b^
*Crumb color*					
L*-crumb	0	68.10 ± 2.40^a^	65.40 ± 1.46^b^	63.71 ± 1.36^c^	56.37 ± 1.74^d^
a*-crumb		−2.80 ± 0.22^c^	−3.24 ± 0.12^d^	−2.39 ± 0.21^b^	−1.11 ± 0.33^a^
b*-crumb		7.49 ± 0.70^d^	17.79 ± 0.86^c^	25.03 ± 0.56^b^	29.08 ± 0.75^a^
ΔE*		–	10.78 ± 0.11^c^	18.12 ± 0.07^b^	24.76 ± 0.32^a^
*Slice morphology*					
2D slice area (cm^2^)	0	2.45 ± 0.07^a^	2.20 ± 0.13^b^	2.25 ± 0.17^b^	2.27 ± 0.09^b^
Mean cell area (mm^2^)		2.48 ± 1.48^a^	1.46 ± 0.58^a^	1.57 ± 0.37^a^	1.48 ± 0.21^a^
Gas cells/cm2		29 ± 14^b^	56 ± 24^a^	65 ± 19^a^	76 ± 13^a^
Porosity (%)		10.70 ± 3.07^b^	14.84 ± 4.17^b^	23.53 ± 11.36^a^	14.67 ± 2.67^b^
*Crumb texture profile analysis*					
Hardness (N)	0	6.7 ± 1.0^c^	13.4 ± 2.9^a^	13.6 ± 4.0^a^	10.5 ± 0.7^b^
	48	31.1 ± 5.5^a^	30.8 ± 13.0^a^	30.2 ± 8.7^a^	17.0 ± 2.2^b^
Hardening rate (%)		464 ± 32^a^	227 ± 37^b^	223 ± 1^b^	162 ± 10^b^
Springiness	0	0.94 ± 0.02^a^	0.90 ± 0.04^b^	0.89 ± 0.01^b^	0.94 ± 0.05^a^
	48	0.91 ± 0.06^a^	0.83 ± 0.07^b^	0.86 ± 0.01^b^	0.83 ± 0.03^b^
Cohesiveness	0	0.87 ± 0.03^a^	0.79 ± 0.07^b^	0.77 ± 0.03^b^	0.80 ± 0.04^b^
	48	0.37 ± 0.08^a^	0.28 ± 0.01^bc^	0.29 ± 0.01^b^	0.26 ± 0.02^c^
Chewiness (N)	0	5.5 ± 1.0^c^	9.5 ± 2.4^a^	9.2 ± 2.4^a^	7.9 ± 0.1^b^
	48	10.6 ± 4.0^a^	7.2 ± 2.2^b^	7.5 ± 1.9^b^	3.6 ± 0.1^c^
Resilience	0	0.65 ± 0.02^a^	0.57 ± 0.07^b^	0.54 ± 0.02^b^	0.57 ± 0.05^b^
	48	0.24 ± 0.05^a^	0.18 ± 0.01^b^	0.18 ± 0.01^b^	0.15 ± 0.02^c^

Data are presented as mean ± standard deviation. Data value of each parameter with different superscript letters, in columns, are significantly different (p < 0.05).

The bread's microstructure was modified (p < 0.05) by the addition of polyphenols (Table 4). The bread volume, represented by the 2D slice area (cm^2^), decreased with the polyphenols added, but no statistical effect was observed with their concentration. Similar trend was observed in the mean cell area (mm^2^), which was lower with added polyphenols. The number of gas cells/cm^2^ and the porosity (%) increased with the polyphenol's incorporation. However, the number of cells was higher as the content of polyphenols increased, but the bread with 0.5% displayed more porosity than the other concentrations. It has been reported that the addition of seaweeds or microalgae into bread formulations, led to a reduction in pore size with a simultaneous increase in the number of cells and porosity, and an overall increase in bread volume [37,38]. However, other authors using polyphenols and observed a decrease in yeast activity, which could explain the reduced 2D slice area found here when incorporating seaweed-extracted polyphenols into the formulation [39]. In samples with polyphenols, the 2D slice area exhibited a statistically significant inverse correlation with the kinetic constant (*r* = −0.9998) and the relative kinetic constant (*r* = −0.9996) of the starch gels during the biochemical digestion.

Textural parameters were measured at time 0 and 48 h. In general, the addition of polyphenols to bread increased (p < 0.05) the hardness on the fresh bread from 6.7 ± 1.0 to 13.6 ± 4.0 N. The increase in crumb hardness could have been due to the crumb being more compact because of, as previously discussed, the inhibition of yeast activity. For samples at 48 h the hardness was around 30 N without significant differences except for bread with 1% of polyphenols which the hardness value decreased until 17.0 ± 2.2 N. The springiness for fresh bread varied between 0.94 and 0.89 and for bread with 48 h the values varied between 0.91 and 0.83. The chewiness increased from 5.5 ± 0.9 to 9.5 ± 2.4 N with the addition of polyphenols in fresh bread. However, the chewiness decreased from 10.6 ± 4.0 to 3.6 ± 0.1 in the bread at 48 h by polyphenols addition. The resilience values decreased significantly with the addition of polyphenols in fresh and at 48 h breads. The hardening rate (%) decreased greatly with the addition of polyphenols which means that polyphenols delayed the starch retrogradation. Polyphenols contain a significant abundance of free hydroxyl groups, capable of establishing hydrogen bonds with starch molecules, thereby inhibiting the reorganization of starch chains through recrystallization [5,40]. Other researchers reported that when some corn and potato starches were replaced by whole red sorghum flour (up to 40% w/w), the hardness decreased [12]. This may be because these authors carried out the trials by substitution while here the trials were by addition. However, the same effect was observed in the springiness values decreasing with the amount of whole red sorghum flour.

## 4 Conclusions

The adsorption of polyphenols on starch was promoted in starch gels regarding native starch because in the gels the available surface area allowing the interactions between starch and polyphenols. On the other hand, the addition of polyphenols in the formation of the starch gel studied by rheology showed a general decrease in the elastic modulus (G') values in the frequency sweep. The results obtained regarding starch digestion indicated that the presence of polyphenols decreased the digestion constant (*k*) and reduced the final concentration of digested starch (C_∞_). The digestion studies were carried out by the biochemical and rheological methods and acceptable agreement between them was found for the evaluation of the relative digestion rate. Finally, polyphenols were added to the starch to make a simplified baking product. The presence of polyphenols increased hardness and chewiness values and decreased the cohesiveness and resilience values. Nonetheless, the crumb hardening during storage was less noticeable, suggesting its potential integration into gluten-free bread formulations to delay bread aging. This could involve process adaptations aimed at mitigating the impact of polyphenols on slice appearance and texture.

## Data availability

Data associated with the study has been deposited into the public Mendeley repository https://doi.org/10.17632/sh8b35h8wm.1.

## Ethics declarations

All authors hereby declare that: Review and approval by an ethics committee was not needed for this study because it did not involve any human subjects, patients, animals, or societal groups.

## Funding

This research was financed by the 10.13039/501100004837Spanish Ministry of Science and Innovation (Project RTI2018-095919-B-C2) and the 10.13039/501100008530European Regional Development Fund (10.13039/501100002924FEDER).

## CRediT authorship contribution statement

**Leticia Montes:** Writing – original draft, Investigation. **Maria Santamaria:** Writing – original draft, Investigation. **Raquel Garzon:** Writing – review & editing, Investigation. **Cristina M. Rosell:** Writing – review & editing, Project administration, Funding acquisition, Conceptualization. **Ramón Moreira:** Writing – review & editing, Project administration, Funding acquisition, Conceptualization.

## Declaration of competing interest

The authors declare that they have no known competing financial interests or personal relationships that could have appeared to influence the work reported in this paper.
